# Bioactivity effects of extracellular matrix proteins on apical papilla cells

**DOI:** 10.1590/1678-7757-2021-0038

**Published:** 2021-09-03

**Authors:** Maria Luísa LEITE, Diana Gabriela SOARES, Giovana ANOVAZZI, MON Filipe Koon Wu, Ester Alves Ferreira BORDINI, Josimeri HEBLING, Carlos Alberto DE SOUZA COSTA

**Affiliations:** 1 Universidade Estadual Paulista Faculdade de Odontologia de Araraquara Departamento de Materiais Odontológicos e Prótese AraraquaraSP Brasil Universidade Estadual Paulista – UNESP, Faculdade de Odontologia de Araraquara, Departamento de Materiais Odontológicos e Prótese, Araraquara, SP, Brasil.; 2 Universidade de São Paulo Faculdade de Odontologia de Bauru Departamento de Dentística, Endodontia e Materiais Odontológicos BauruSP Brasil Universidade de São Paulo, Faculdade de Odontologia de Bauru, Departamento de Dentística, Endodontia e Materiais Odontológicos, Bauru, SP, Brasil.; 3 Universidade Estadual Paulista Faculdade de Odontologia de Araraquara Departamento de Morfologia e Clínica Infantil AraraquaraSP Brasil Universidade Estadual Paulista – UNESP, Faculdade de Odontologia de Araraquara, Departamento de Morfologia e Clínica Infantil, Araraquara, SP, Brasil.; 4 Universidade Estadual Paulista Faculdade de Odontologia de Araraquara Departamento de Fisiologia e Patologia AraraquaraSP Brasil Universidade Estadual Paulista – UNESP, Faculdade de Odontologia de Araraquara, Departamento de Fisiologia e Patologia, Araraquara, SP, Brasil.

**Keywords:** Guided tissue regeneration, Dental pulp, Fibronectin, Laminin, Collagen

## Abstract

Potent signaling agents stimulate and guide pulp tissue regeneration, especially in endodontic treatment of teeth with incomplete root formation.

**Objective:**

This study evaluated the bioactive properties of low concentrations of extracellular matrix proteins on human apical papilla cells (hAPCs).

**Methodology:**

Different concentrations (1, 5, and 10 µg/mL) of fibronectin (FN), laminin (LM), and type I collagen (COL) were applied to the bottom of non-treated wells of sterilized 96-well plates. Non-treated and pre-treated wells were used as negative (NC) and positive (PC) controls. After seeding the hAPCs (5×10^3^ cells/well) on the different substrates, we assessed the following parameters: adhesion, proliferation, spreading, total collagen/type I collagen synthesis and gene expression (ITGA5, ITGAV, COL1A1, COL3A1) (ANOVA/Tukey; α=0.05).

**Results:**

We observed greater attachment potential for cells on the FN substrate, with the effect depending on concentration. Concentrations of 5 and 10 µg/mL of FN yielded the highest cell proliferation, spreading and collagen synthesis values with 10 µg/mL concentration increasing the ITGA5, ITGAV, and COL1A1 expression compared with PC. LM (5 and 10 µg/mL) showed higher bioactivity values than NC, but those were lower than PC, and COL showed no bioactivity at all.

**Conclusion:**

We conclude that FN at 10 µg/mL concentration exerted the most intense bioactive effects on hAPCs.

## Introduction

The concepts of tissue engineering were introduced to Dentistry in the last decade giving special attention to inducting pulp regeneration.^1-4^ The principles of regenerative endodontics are four: 1. disinfection and detoxification of root canals; 2. use of biomaterials as scaffold for cell adhesion, proliferation, and differentiation; 3. presence of cells with potential to regenerate a new tissue similar to the original; and 4. use of signaling agents to induce cell migration and enhance the bioactive action of the biomaterial.^5,6^

Two strategies emerged as the most promising therapies for tissue regeneration: cell approaches and cell-free approaches.^7,8^ The first involves implanting pre-cultured cells associated with a biomaterial at the site of injury. The bioactive materials provide a porous three-dimensional structure and act as a temporary extracellular matrix where attached cells can grow to regenerate the tissue. The second, cell-free approaches, involve the use of biomaterials along potent signaling agents. When put into the injury site, these agents stimulate cell migration towards the site of interest, as well as their proliferation and differentiation, favoring tissue healing.^7,8^

Cell-free therapies seem to be interesting for teeth with incomplete root formation. A histopathological study of rat teeth with periapical lesion demonstrated a viable apical papilla was maintained even after 90 days of pulp necrosis.^9^ The apical papilla is known to have a rich source of stem cells. Therefore, biomaterials associated with potent signaling agents could be used to attract these cells into the root canal. These biomaterials should also promote adhesion, proliferation and differentiation of the stem cells along the entire length of the root canal, so they can synthetize new pulp-like tissue.^1^

Fibronectin (FN), laminin (LM) and type I collagen (COL) – examples of extracellular matrix proteins (ECMp) – have bioactive properties and were proposed as chemotactic and inducing agents, since scientists have described their proprieties in several biomedical fields.^10-15^ Previous studies showed FN, either associated with other proteins, or not, can mediate cell adhesion, migration, proliferation, and differentiation; while also helping in tissue formation, remodeling, repair and regeneration.^11,13,15^ LM is a complex structure that also regulates cell adhesion, migration and proliferation.^10,12^ LM aids in maintaining cellular functions especially in the processes of reeptelialization and angiogenesis.^10^ COL is a common protein that surround cells throughout the body, with over 90% of this protein being type I. COL can be used to mimic the natural cell environment, and under specific conditions, is effective in promoting stem cell differentiation and growth.^13,16^

One study characterized the dehydrated human umbilical cord (DHUc) in terms of tissue composition and evaluated its *in vitro* and *in vivo* effects.^14^ The authors observed the presence of COL, hyaluronic acid, FN and LM, among other proteins, which included growth factors, inflammatory modulators and cell signaling regulators in the DHUc. They observed increased migration of fibroblasts, and higher level of mesenchymal stem cell proliferation, associated with induction of angiogenic potential *in vitro* depending on concentration when cells were treated with DHUc. When implanted into subcutaneous tissue of rats, this biomaterial proved to be biocompatible and biodegradable.^14^Researchers have reported decellularized human dental pulp, which maintains several proteins of this specialized connective tissue, like COL, FN and LN, is a suitable scaffold to mimic the complexity of the dental pulp extracellular matrix.^17,18^ Decellularized biological scaffolds allow stem cells from apical papilla^17^ as well as human dental pulp stem cells^18^ to attach and proliferate while being stimulated to differentiate into odontoblast-like cells close to the dentin substrate.^18^

Despite the extracellular matrix proteins role on pulp regeneration and formation of a new pulp-like tissue, the data concerning the influence of FN, LN and COL on the metabolism and function of human apical papilla cells (hAPCs) – especially when young teeth with incomplete root formation have lost their vitality – is scarce. Thus, this *in vitro* study assessed the effects of FN, LM and COL on the adhesion, proliferation and spreading of hAPCs as well as on the potential of these cells to synthesize collagen and express genes related with pulp regeneration.

## Methodology

Firstly, approval of the research was obtained from the Research Ethics Committee of Araraquara School of Dentistry/UNESP, São Paulo, Brazil (protocol Nº. 80806617.3.0000.5416) as well as signed terms of informed consent from patients and their guardians, according to the Declaration of Helsinki. Subsequently the hAPCs were obtained from the apical papilla of four healthy third molars with incomplete root formation. These teeth were provided by volunteers aged 16 to 18 years old, of both sexes, who were patients at the surgery clinic. The teeth were extracted for orthodontic reasons. To obtain the primary culture of hAPCs, the extracted teeth were immediately immersed in Minimum Essential Medium Eagle Apha-culture medium (α-MEM; GIBCO, Carlsbad, CA, USA) supplemented with antibiotic and antifungal (100 U/mL penicillin, 100 g/mL streptomycin, and 0.25 g/mL amphotericin; GIBCO) at 4ºC, for 1 hour. In a procedure performed in a biosafety cabinet (Bio Protector 12 Plus; VECO, Campinas, SP, Brazil), the papilla was sectioned with a sterile scalpel blade, at the apical limit of the root. This tissue was transferred to a 1.5 mL tube containing phosphate buffered saline (PBS 1X; GIBCO), followed by mechanical disintegration with sterile surgical scissors. Then, the apical papilla fragments were incubated in α-MEM containing 3 mg/mL type I collagenase (Sigma-Aldrich, Saint Louis, MO, USA) at 37°C and 5% CO_2_ for 2 h. After this period, the cells were centrifuged, washed with PBS 1X, and later seeded in wells of a 6-well plate (Corning, Tewksbury, MA, USA); for the seeding, we used α-MEM supplement with 10% fetal bovine serum (FBS), 100 U/mL penicillin, 100 g/mL streptomycin, and 0.25 g/mL amphotericin (GIBCO). After 3 h incubation, the supernatant was discarded and the cells that remained attached to the bottom of the wells were sub-cultured in 100×15 mm petri dishes (Corning). Cells at passage 3 to 5 were used in the experiments.

Fibronectin (2.0 mg/mL, bovine plasma; Sigma-Aldrich), laminin (1 mg/mL, Engelbreth-Holm-Swarm lathyritic mouse tumor; Santa Cruz Biotechnology), and type I collagen (3.67 mg/mL, rat trial; Corning, Bedford, MA, USA) were used in this study. These ECMps were diluted in PBS 1X to obtain concentrations of 1, 5 and 10 µg/mL that were applied (50 µL) to the bottom of wells without pretreatment of sterilized polystyrene 96-well plates (Corning; Product Number: 3370). The 96-well plates were then centrifuged at 1500 rpm at 4ºC for 10 min. After keeping the plates at 4ºC for 18 h, the remaining material was aspirated. These plates were incubated with 0.5% bovine serum albumin (BSA; Santa Cruz Biotechnology) for 10 min to block the uncoated surface,^19,20^ followed by washing with PBS 1X at 37ºC. All these steps were performed for both negative (NC) and positive (PC) control groups. In the NC, PBS solution without ECMp was applied to the bottom of non-pretreated wells of sterilized polystyrene 96-well plates (Corning; Product Number: 3370). In the PC, APBS without ECMp solution was applied to the bottom of wells of polystyrene 96-well plates that were pre-treated by the manufacturer for cell culture purpose (Corning; Product Number: 3395). Table 1 shows all groups established according to the polystyrene 96-well plates used, as well as the type and concentration of ECMp.

**Table 1:** Experimental and control groups according to type of polystyrene 96-well plate and type and concentration of extracellular matrix protein (ECMp)

Groups	Pre-treated wells by the manufacture	ECMp	Concentration (µg/mL)
NC (negative control)	-	-	0
PC (positive control)	+	-	0
FN1	-	Fibronectin	1
FN5	-	Fibronectin	5
FN10	-	Fibronectin	10
LM1	-	Laminin	1
LM5	-	Laminin	5
LM10	-	Laminin	10
COL1	-	Type I collagen	1
COL5	-	Type I collagen	5
COL10	-	Type I collagen	10

After performing the treatments of wells with ECMp, the hAPCs (5×10^3^cells/well) were seeded in the respective wells and incubated at 37ºC and 5% CO_2_ for up to 5 days, with the culture medium being changed every 48 h. Then, the bioactivity parameters were evaluated. All assays were performed twice to ensure the data reproducibility.

Cell adhesion: Since the non-treated 96-well plates did not allow cell attachment on the bottom of the wells, the attachment potential of the microplate treated with different concentrations of ECMp was assessed. For this purpose, protocols of dynamic systems, in which microchannels are treated with proteins to induce cell chemotaxis,^19-21^ were adapted to this study. After 24 h of applying cell suspensions to the plate wells (n=6), the hAPCs capable of migrating and attaching to the bottom of wells were evaluated under a light microscope. For this purpose, the cells were fixed with 2.5% glutaraldehyde for 2 h, washed with distilled water and stained with 0.1% crystal violet for 15 min. For quantitative analysis, photographs (1 field from the central region of each well of the microplate; 10× magnification) were obtained with a camera (Olympus C5060, Miami, FL, USA) coupled to a microscope (Olympus BX51, Miami, FL, USA). The images were evaluated using Image J 1.45S software (Wayne Rasband, National Institutes of Health, USA) to count the number of cells on each image.

Cell proliferation: This analysis was performed by the MTT assay (n=6). After intervals of 1, 3 and 5 days in α-MEM, cells were incubated for 4 h in α-MEM supplemented with 10% MTT solution (5 mg/mL; Sigma-Aldrich).^22^ Then, the formazan crystals produced were dissolved in acidified isopropanol and absorbance of the resulting solution was measured at 570 nm (Synergy H1, Biotek, Winooski, VT, USA). The mean absorbance value obtained in the negative control group over the 1-day period was considered 100% cell proliferation. This parameter was used to determine the percentage of viability for the other groups.

Cell spreading: For this analysis (n=4), after intervals of 1, 3 and 5 days in α-MEM, the cells were fixed in 4% paraformaldehyde, permeabilized in 0.1% Triton X-100 (Sigma-Aldrich) and incubated with Actin Red 555 probe (Life Technologies; Grand Island, NY, USA) in 2% BSA (1:20) to detect the actin filaments. After washing the cells with PBS, they were incubated with Hoescht (1:5000; Invitrogen, Carlsbad, CA, USA) for 15 min for nuclear counter-staining.^23^ The F-actin assay was then analyzed using a fluorescence microscope (Leica DM 5500B).

Total collagen synthesis: The cells were cultured for 5 days in α-MEM without FBS, and the culture medium was collected and replaced every 48 h. The pool of collected culture medium of each sample was stored at -20ºC until the Sirius Red assay was performed (n=6). For this purpose, the culture was transferred to 1.5 mL tubes containing Direct Red solution in saturated picric acid (0.1%), and then incubated for 1 hour, under agitation at 400 rpm, in a dry bath at 25°C. The tubes were centrifuged, the supernatant was discarded, and 0.01 M hydrochloric acid was added. The tubes were centrifuged again, the supernatant was discarded, followed by the addition of 0.5 M sodium hydroxide to solubilize the precipitated material.^24^ The resulting solution from each sample was subjected to absorbance analysis in a spectrophotometer at 555 nm (Synergy H1).^24^ The percentage of total collagen synthesis for each sample was calculated based on the mean absorbance values of the negative control group.

Type I collagen synthesis: The medium pool collected in the previous analysis (n=6) was also used to evaluate the type I collagen synthesis (COL-I), which was detected by enzyme-linked immunosorbent (ELISA) assay performed with a standardized kit (Duoset human COL-I - R&D Systems, Minneapolis, MN, USA), according to manufacturer’s instructions. For this purpose, 96-well plates for ELISA (Corning) were treated with 4 µg/mL of Capture Antibody and incubated overnight. After this period, the wells were incubated with Reagent Diluent 1X for 1 hour. Then, standardized aliquots of the samples and standard curve points were applied and incubated for 2 h. After this, the wells were maintained with 100 ng/mL Detection Solution for 2 h, followed by incubation in dark room, with 2.5% Streptavidin-HRP, for 20 min. The wells were washed with Wash Solution 1X between all steps described. Finally, Substrate Solution (color reagent A + color regent B; 1:1) was applied and after 20 minutes, the Stop Solution was added. The absorbance reading was performed at a wavelength of 450 nm in a spectrophotometer (Synergy H1). The concentration of type I collagen for each sample was determined according to the standard curve. The percentage of type I collagen synthesis for each sample was calculated based on the average concentration values, in ng/mL of the positive control group.

**Figure 1:**
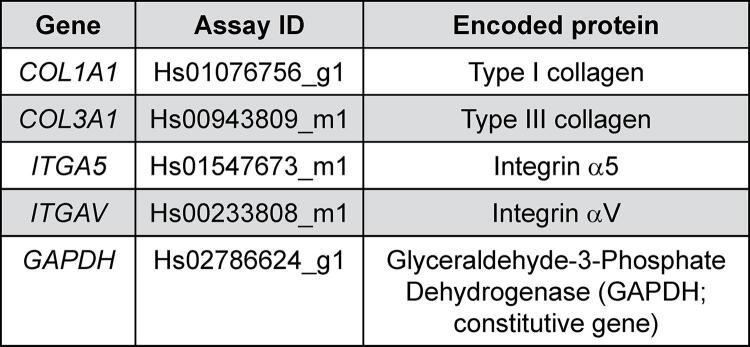
TaqMan assays used to analyze gene expression

Reverse transcription followed by quantitative polymerase chain reaction (RT-qPCR): After incubating the cells in α-MEM for 5 days (n=4), the RNAqueous kit (Ambion, Grand Island, NY, USA) was used to isolate the RNA in accordance with the manufacturer’s instructions. To obtain sufficient RNA for the reaction, a pool of cells obtained from two wells was used for each group. Then, 500 ng of total RNA associated with random hexamer primers and Moloney leukemia virus reverse transcriptase were used to determine the cDNA synthesis (n=4), according to the instructions of the High Capacity RT kit supplier (Applied Biosystems, Foster City, CA, USA). For the gene expression of ITGA5, ITGAV, COL1A1 and COL3A1 (Figure 1) by qPCR was performed, using pre-designed sets of primers and probes (Gene expression assays, Applied Biosystems) to detect target genes by the TaqMan system (TaqMan Universal PCR Master Mix, Applied Biosystems) with the StepOne Plus equipment (Applied Biosystems). In addition, the cycling conditions optimized by the manufacturer were used, and the cycle threshold (CT) values for each sample were calculated using the thermal cycler software and analyzed using the 2^CT^ method. The results were normalized according to the constitutive gene expression (GAPDH) and expressed as a fold change in relation to the positive control.

The data from cell adhesion, viability, total protein synthesis, type I collagen synthesis, and RT-qPCR assays were evaluated for adherence to the normal curve (Shapiro-Wilk test, p>0.05) and homoscedasticity (Levene test, p>0.05). Then, the data were submitted to the one-way or two-way ANOVA test, followed by the Tukey post-hoc test by means of SPSS 20.0 software (SPSS Inc., Chicago, IL, USA). All statistical inferences were based on the 5% level of significance. The DSS Research calculator was used to calculate the statistical power of the samples, according to Kim^25^ (2016), which showed a power level > 95.0% for each analysis. The cell spreading images were analyzed descriptively.

## Results

**Figure 2:**
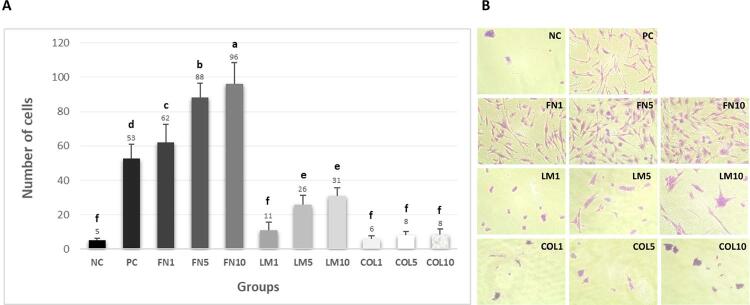
Cell adhesion assay. (A) Mean and standard deviation of the number of cells adhered to the bottom plate treated according to the group. Different letters demonstrate a significant difference between groups (n=6; one-way ANOVA/Tukey tests; α=0.05). (B) Representative images of the cell adhesion

Cell adhesion: As demonstrated in Figure 2A and B, the highest attachment potential occurred in Group FN10, followed by Groups FN5, FN1, PC, LM10, and LM5 (p<0.05). No significant difference was observed between Groups LM1, COL1, COL5, and COL10 and Group NC (p>0.05).

**Figure 3:**
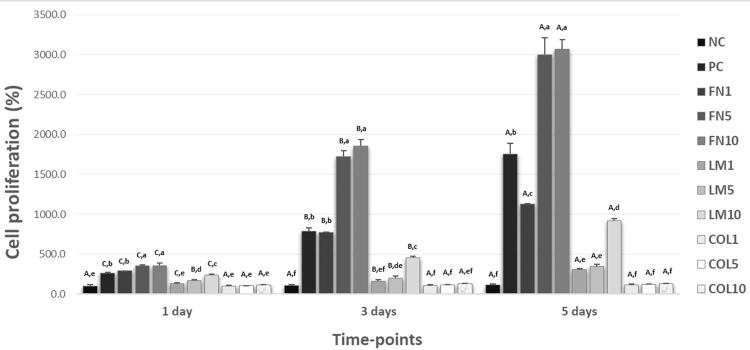
Mean and standard deviation of cell proliferation values (n=6; two-way ANOVA/Tukey tests; α=0.05). Different capital letters indicate a significant difference between the time-points for each group. Different lowercase letters demonstrate a significant difference between groups in each time-point

Cell proliferation: Figure 3 shows increased cell proliferation occurred over time-points for all groups (p<0.05), except for those treated with type I collagen and Group NC (p>0.05). The best cell viability result was observed in the groups treated with 5 or 10 µg/mL of FN after intervals of 1, 3 and 5 days (p<0.05). At the last time-point, this increase was 30x higher when compared with Group NC, 1.7x higher than in Group PC, and 2.7x higher than the group treated with 10 µg/mL LM. The group FN1 also showed higher cell viability values than the group LM10 in all time-points (p<0.05).

**Figure 4:**
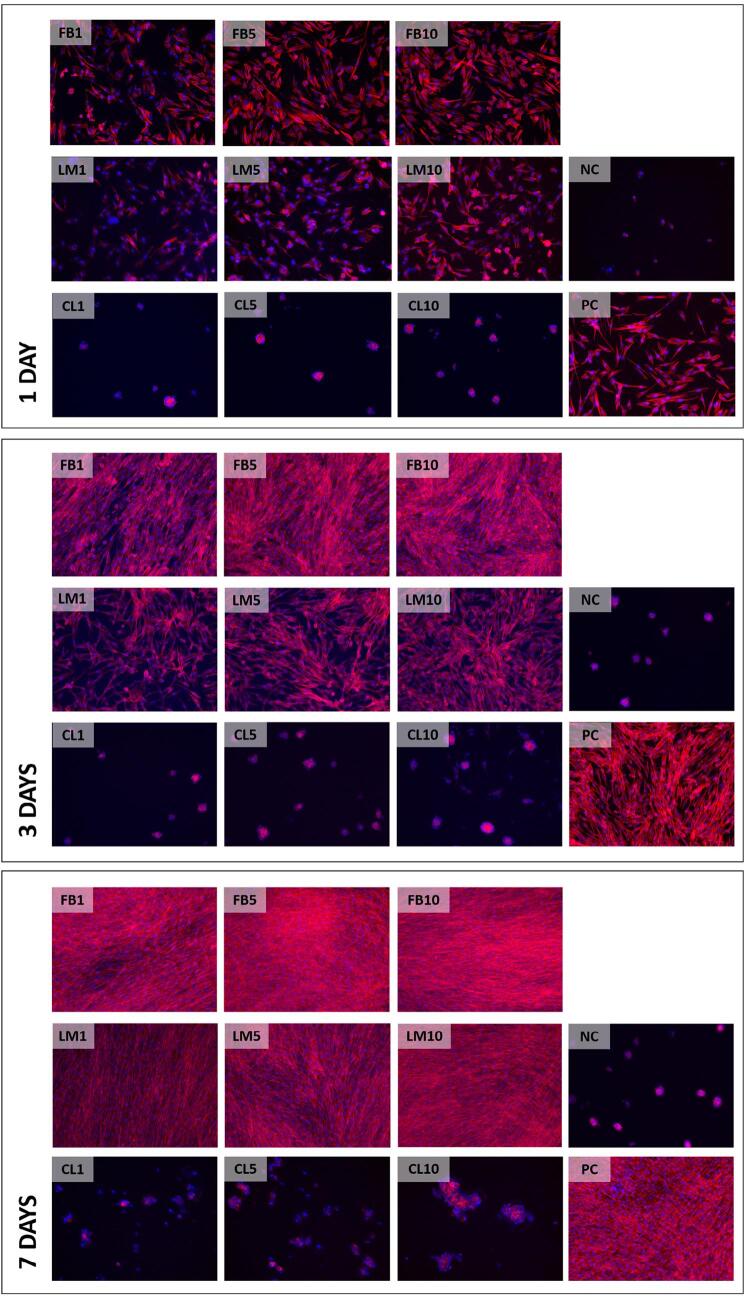
Representative images of F-actin assay (10×). Red fluorescence indicates the actin filaments. Blue fluorescence indicates the cell nucleus

Cell spreading: Figure 4 displays how cells seeded on the bottom of the wells treated with 5 or 10 µg/mL of FN had better spreading on the substrate and a higher rate of proliferation over the time-points. In the groups treated with COL and in Group NC, lower numbers of cells organized in clusters exhibiting contracted cytoskeletons remained attached to the wells bottom.

**Figure 5:**
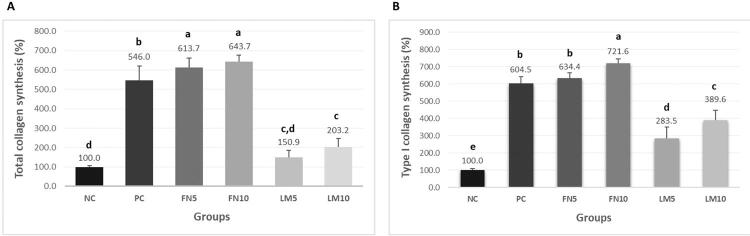
Mean and standard deviation of total collagen (A) and type I collagen (B) synthesis values (n=6; one-way ANOVA/Tukey tests; α=0.05). Different letters demonstrate a significant difference between groups

Total collagen synthesis: Figure 5A shows increase in the total collagen synthesis occurred in Groups FN10 and FN5, followed by Groups PC and LM10 in comparison with Group NC (p<0.05).

Type I collagen synthesis: Figure 5B demonstrates that more type I collagen was synthesized in Group FN10 (p<0.05), followed by Groups FN5 and PC (p<0.05), which showed no significant difference between them (p>0.05). Groups LM5 and LM10 showed lower values of type I collagen synthesis in comparison with Group PC (p<0.05), but higher than those observed for Group NC (p<0.05).

**Figure 6:**
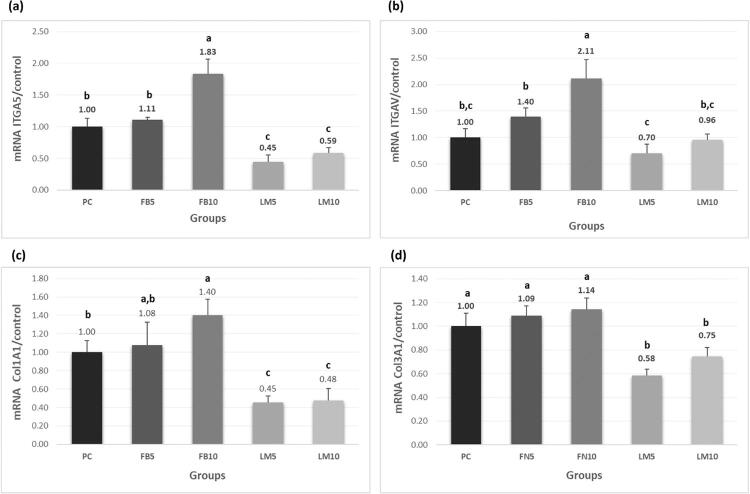
Mean and standard deviation of mRNA gene expression values of the ITGA5 (A), ITGAV (B), COL1A1 (C) and COL3A1 (D) markers (n=4; one-way ANOVA/Tukey tests; α=0.05). Different letters demonstrate a significant difference between groups

RT-qPCR: Figure 6A-D shows higher levels of ITGA5, ITGAV, and COL1A1 gene expression occurred in Group FN10 in comparison with Group PC (p<0.05). However, FN10 and PC groups COL3A1 gene expression had no significant difference (p>0.05). Group FN5 showed no statistical difference when compared with Group PC for all evaluated genes (p>0.05), and Groups LM5 and LM10 demonstrated lower values of gene expression than Group PC (p<0.05).

## Discussion

The application of tissue engineering concepts for the purpose of pulp tissue regeneration has been relevant, especially in endodontic treatment of teeth with incomplete root formation. The thin and fragile walls of the root canal, associated with the short length of the root, make these teeth susceptible to fracturing under the forces to which they are submitted.^7,26^ Conservative treatments that aim to stimulate cell migration from apical papilla into the root canal and allow the synthesis of a new pulp-like tissue seem to be an interesting alternative that would allow the return of tooth vitality and continuity of root formation. For this purpose, the use of a potent signaling agent is crucial to stimulate and guide tissue regeneration.^5,6^ Studies have shown extracellular matrix proteins in concentrations between 0.5 to 100 µg/mL can act as a bioactive agent on cells from different sources.^27,30^ Therefore, in the present study, we assessed the biological activities of low concentrations of ECMp (fibronectin, laminin and type I collagen) on hAPCs.

Type I collagen can regulate the cell behavior by specific signals and trigger several biological activities essential for tissue development and homeostasis.^11^ However, the different concentrations of COL tested in this investigation showed no bioactive potential on the hAPCs. As observed in the negative control, only a few cells remained attached to the COL over the time-points. These cells, which exhibited cytoplasm contraction, were organized in clusters. Parisi, et al.^15^ (2020) reported that adhesion represents both the interaction between cells and the substrate on which they were seeded, and the interaction and communication among surrounding cells, which activate the mechanisms of proliferation and differentiation. These authors showed that lack of cell adhesion to the substrate and interaction with surrounding cells resulted in apoptosis.^15^ Therefore, according to the data obtained in the present study, and given the relevant role of collagen in different cell functions,^4,11,31-33^ we suggest that the concentrations of type I collagen tested on the hAPCs were insufficient to stimulate cell adhesion, proliferation and spreading. As the study aimed to evaluate and compare the bioactive effects of low dosages of ECMp, the same concentrations of FN, LM and COL were evaluated on hAPCs. However, based upon the negative data obtained for COL, we recommend the assessment of higher concentrations of this type of ECMp on hAPCs in future studies.

Although the cell attachment of LM was lower in comparison with the positive control, we observed a dose-response effect on hAPCs when using different concentrations of this protein. The assays using 5 and 10 µg/mL of LM yielded greater cell adhesion, proliferation and spreading in comparison with the negative control. A recent study showed LM expression in blood vessels regulated the viability and migration of oligodendrocyte precursor cells via β1 integrin-focal adhesion kinase (FAK).^30^ In addition, Liu, et al.^34^ (2017) demonstrated *in vivo* that 316L stainless steel treated with stromal cell-derived factor-1α (SDF-1α)/laminin had increased biocompatibility and improved the healing of vascular lesions. Enhanced adhesion, migration and proliferation of human limbal epithelial stem cells was also demonstrated in tests with specific isoforms of LM incorporated into a fibrin-based hydrogel.^12^ These positive effects of LM have been shown on Schwann cells^33^ as well as on hepatocytes and endothelial cells.^11,13^ Therefore, the results of the present study, in which LM stimulated the adhesion, proliferation and spreading on hAPCs, corroborate with these data previously reported by several researchers.^11,12,13,33,35^

This study also showed that the bioactive potential of 1, 5 and 10 µg/mL of FN was concentration-dependent and promoted strong adhesion on hAPCs compared with the positive control and other experimental groups. Additionally, concentrations of 5 and 10 µg/mL of FN stimulated the highest proliferation and spreading of hAPCs at all time-points. These data are in line with a previous investigation in which the authors demonstrated an experimental hydrogel containing FN exerted higher chemotactic effect on NIH 3T3 fibroblasts than the same hydrogel containing LM.^32^ The role of FN in inducing cell migration and differentiation on dental pulp cells is already known.^36,37^ Chang, et al.^38^ (2016) evaluated the effect of titanium surfaces treated with glow discharge plasma followed by FN adsorption on MG-63 osteoblast-like cells. The authors observed that the modified titanium surfaces enhanced cell adhesion, migration and proliferation. Matsui, et al.^28^ (2015) also reported an increased cell adhesion in situ when FN-coated hydroxyapatite was assessed under a defined experimental condition. Amplified cell migration, adhesion, spreading and proliferation occurred when periodontal ligament cells were treated with 5 or 10 µg/mL of fibronectin.^27^ More recently, Parisi, et al.^15^ (2020) demonstrated that high concentrations of fluid containing FN allowed rapid deposition of this protein on biomaterials, what improved the adhesion and proliferation of cells on them.

Taking into consideration all the interesting data concerning the positive effects of FN and LM on different cell lines and based upon the preliminary results obtained in this *in vitro* study, the two highest concentrations of FN and LM were selected for the subsequent experiments. In the present investigation, only the use of 10 µg/mL of FN significantly increased the synthesis of total collagen, as well as gene expression and synthesis of type I collagen in comparison with the positive control. Despite the tendency towards increase in the expression of type III collagen, there was no significant difference between FN10 and the positive control. Collagen is an abundant protein in the extracellular matrix of pulp tissue, with type I and type III collagen representing about 56% and 41%, respectively, of the organic content.^31^ Previous studies described the presence of FN in tissue engineering strategies as an approach to induce collagen synthesis and stimulate tissue regeneration.^33,39^ Collagen and other proteins present in the extracellular matrix have interaction sites with different specificities for cell membrane receptors, turning the cells capable of binding and triggering distinct biological activities related to development, homeostasis and formation of a new tissue.^11^

To better understand the mechanism of action of FN and LM on hAPCs, we also evaluated the gene expression of α5 integrin (ITGA5) and αv integrin (ITGAV); these genes are related to cell migration and adhesion.^25,40-42^ The interaction of α5β1 and αvβ3 integrins and the role of these membrane receptors in the detection of extra-cellular matrix proteins have been demonstrated.^42^ Although these integrins have distinct biological intracellular mechanisms, both act in the detection processes of specific ligands that trigger particular cell signaling related to the projection mechanisms and directional migration of the cytoskeleton. The joint action of integrins allows cells to adapt to the microenvironment and perform their functions in tissue remodeling and regeneration.^44^ In the present study, we observed higher gene expression of ITGA5 and ITGAV only for FN10 in comparison with the positive control (p<0.05). In a study conducted with a parental fibroblast lineage, Spoerri, et al.^43^ (2020) observed that when the α5β1 and αVβ3 integrins were activated by PAR1 and PAR3 proteases, via Gβγ and PI3K signaling, they changed their conformation, thereby enhancing their affinity to FN. Thus, the cell adhesion established was strengthened by means of specific biological signals mediated by PARs via Gα13, Gαi, ROCK and Src. Also, the authors reported the cell migration and proliferation were accelerated after cell adhesion was triggered.^33^ The effect of proteins that contain a functional domain of FN and LM was also observed in a primary culture of human keratinocytes.^44^ When these cells were subjected to GTPase inhibitors in the presence of FN and LM-mimic surfaces, the modulation of cell adhesion and migration was dependent on the Rac1 and Rho pathways.^44^ Nevertheless, in the presence of the α5β1 integrin receptor, FN-mimic was capable of inducing cell migration and increasing keratinocyte adhesion, whereas under these conditions, LM-mimic was unable to stimulate cell motility.^44^ Therefore, we could suggest that the type and concentration of the ECMp influence the cell adhesion and migration, and at least partly explain the strong bioactive potential of 10 µg/mL of FN on the HAPCs observed in the present study.

Overall, the need to replace the traditional endodontic treatment of teeth with incomplete root formation (which aims to induce apexification and later fill the root canal with definitive materials), has led to the hypothesis that associating the bioactive properties of FN with those of an interesting biomaterial can regenerate the lost pulp tissue. Previous studies have shown that the association of extracellular matrix proteins with scaffolds approximated the conditions of the *in vivo* environment.^13,29,33^Thus, they mediated the mechanical-chemical signals in the processes of adhesion, migration, proliferation and differentiation of various cell types, and in the synthesis of a new matrix.^13,29,33^The results obtained in the present study cannot be directly extrapolated to clinical situations. However, in spite of the limitations of this laboratory investigation, it seems appropriate to conduct further studies to assess techniques using the cell-free approaches proposed by Galler and Widbiller^7^ (2020). In these, the root canals may be filled with biomaterials containing dosages of FN fibronectin capable of stimulating the migration of cells from apical papilla and allowing new pulp-like tissue formation.

## Conclusions

According to the methodologies used in the present study, we conclude that 10 µg/mL of fibronectin could act as a potent bioactive agent for human apical papilla cells by inducing cell adhesion, proliferation, spreading and collagen synthesis.
